# Determining intention, fast food consumption and their related factors among university students by using a behavior change theory

**DOI:** 10.1186/s12889-022-12696-x

**Published:** 2022-02-15

**Authors:** Alireza Didarloo, Surur Khalili, Ahmad Ali Aghapour, Fatemeh Moghaddam-Tabrizi, Seyed Mortaza Mousavi

**Affiliations:** 1 Social Determinants of Health Research Center, Clinical Research Institute, Urmia University of Medical Sciences, The Province of Western Azarbaijan, Urmia, 5756115198 Iran; 2 Faculty of Health, Urmia University of Medical Sciences, the Province of Western Azarbaijan, Urmia, 5756115198 Iran; 3 Reproductive Health Research Center, Urmia University of Medical Sciences, the Province of Western Azarbaijan, Urmia, 5756115198 Iran; 4 Faculty of Paramedical Sciences, Urmia University of Medical Sciences, the Province of Western Azarbaijan, Urmia, 5756115198 Iran; 5 Department of Paramedical Science, School of Paramedical Sciences, Urmia University of Medical Sciences, Urmia, Iran

**Keywords:** Fast food, Predictor, Student, Theory of planned behavior, Iran

## Abstract

**Background:**

Today, with the advancement of science, technology and industry, people’s lifestyles such as the pattern of people’s food, have changed from traditional foods to fast foods. The aim of this survey was to examine and identify factors influencing intent to use fast foods and behavior of fast food intake among students based on the theory of planned behavior (TPB).

**Methods:**

A cross-sectional study was conducted among 229 university students. The study sample was selected and entered to the study using stratified random sampling method. Data were collected using a four-part questionnaire including Participants’ characteristics, knowledge, the TPB variables, and fast food consumption behavior. The study data were analyzed in SPSS software (version 16.0) using descriptive statistics (frequencies, Means, and Standard Deviation) and inferential statistics (t-test, Chi-square, correlation coefficient and multiple regressions).

**Results:**

The monthly frequency of fast food consumption among students was reported 2.7 times. The TPB explained 35, 23% variance of intent to use fast food and behavior of fast food intake, respectively. Among the TPB variables, knowledge (*r* = .340, *p* < 0.001) and subjective norm (*r* = .318, *p* < 0.001) were known as important predictors of intention to consume fast foods - In addition, based on regression analyses, intention (*r* = .215, *p* < 0.05), perceived behavioral control (*r* = .205, *p* < 0.05), and knowledge (*r* = .127, *p* < 0.05) were related to fast food consumption, and these relationships were statistically significant.

**Conclusions:**

The current study showed that the TPB is a good theory in predicting intent to use fast food and the actual behavior. It is supposed that health educators use from the present study results in designing appropriate interventions to improve nutritional status of students.

**Supplementary Information:**

The online version contains supplementary material available at 10.1186/s12889-022-12696-x.

## Background

Over the past few decades, non-communicable diseases such as eczema, asthma, cancer, type 2 diabetes, obesity, etc. have increased in developed countries [1, 2]. Also, these diseases are more prevalent with increasing urbanization in developing countries [3–5]. The occurrence of many non-communicable diseases is related to diet [6]. Food habits are rooted from cultural, environmental, economic, social and religious factors. An effective factor in the development of chronic diseases is lifestyle, dietary patterns and habits. Inappropriate food habits and unhealthy environments have increased the incidence of non-communicable diseases in the world [7, 8].

Many developing countries with a tendency towards Western dietary culture go away from traditional and local diets [6]. Healthy foods with nutrients have been replaced by new foods called fast foods [9]. Fast food is the food prepared and consumed outside and often in fast food restaurants [10]. Fast food is often highly processed and prepared in an industrial fashion, i.e., with standard ingredients and methodical and standardized cooking and production methods [10]. In fast food, vitamins, minerals, fiber and amino acids are low or absent but energy is high [9]. Fast food consumption has increased dramatically in the last 30 years in European and American countries [11].

Previous studies reported patterns of inappropriate and harmful food consumption in Iranian children and adolescents [12, 13]. Most fast food customers are adolescents and youth, as these products are quickly and easily produced and relatively inexpensive [14]. One Iranian study shows that 51% of children eat inappropriate snacks and drinks over a week [15]. It is also reported that adults today consume fast food more than previous generations [16]. Faqih and Anousheh reported that 20% of adolescents and 10% of adults consumed sandwiches 3 or more times a week [17].

According to two studies, children and adolescents who consume fast food have received more energy, saturated fat, sodium, carbohydrates and more sugar than their peers, but they have less fiber, vitamin A and C, and less fruit and vegetables [18, 19]. Also, because of the use of oils to fry these foods at high temperatures, these types of foods may contain toxic and inappropriate substances that threaten the health of consumers [20].

In a study in the United States on young people between 13 and 17 years old, it was found that there is a significant relationship between weight gain and obesity with pre-prepared foods [21]. According to the Center for Disease Control and Prevention (2007–2008), 17% of children aged 2 to 19 years and 34% of those aged 20 years and older were obese [22]. Many Health problems were caused by human health behavior(e.g. exercising regularly, eating a balanced diet, and obtaining necessary inoculations, etc.) and studying behavior change theories/models provides a good insight into the causes and ways of preventing these problems [23]. One of these theories is the Theory of Planned Behavior (TPB), which is a developed form of the Theory of reasoned action (TRA), and describes a healthy behavior that is not fully under the control of a person [24]. This theory can successfully predict eating habits and behaviors, and recently this theory has received considerable attention from researchers in identifying norms and beliefs related to the use of fast food [25].

Based on the TPB, intention to conduct a behavior with following three concepts is controlled: 1. Attitudes (positive and negative evaluation of a behavior), 2. Subjective norms (social pressure received from peers, family, health care providers for doing or not doing a given health behavior), 3. Perceived behavior control (This refers to a person’s perception of the ease or difficulty of performing the behavior of interest.) [26–28].

The TPB has been tested on different behaviors such as healthy food choice [24], physical activity [29], and fast food consumption [30]. For instance, the study conducted by Seo et al. showed that fast food consumption behavior was significantly associated with behavioral intention and perceived behavioral control. In addition, their findings highlighted that behavioral intention was significantly related to subjective norm and perceived behavioral control [28].

Given that our study population has cultural diversity and nutritional behaviors different from the societies of other countries and According to the mentioned materials, the researchers decided to test the study with the aim of investigating and explaining the intention and behavior of fast food consumption and their related factors based on the TPB among Urmia University of Medical Sciences students. The results of this study will increase the awareness and knowledge about fast food and, in addition, its results can be used in research, hospitals and healthcare settings.

## Methods

### Subjects

This cross-sectional study was performed on students of Urmia University of Medical Sciences located in northwest Iran in academic year of 2018–2019. The inclusion criteria for the study are females and males who studied at Urmia University of Medical Sciences, and students’ voluntary participation in the study and obtaining written consent from the students and University principals for the students’ participation in the study. The lack of willingness to continue participating in the study and not signing the informed consent form were considered as exclusion criteria.

According to the results of the study of Yar Mohammadi and et al. [31], with a 95% confidence interval and an error of 0.05, using the formula for estimating the proportion in society, taking into account the 10% drop rate, sample size was estimated 330students. A randomized stratified sampling method was used to select the study samples. The study sample was randomly selected from each of the strata based on the share of the total sample.

### Questionnaire

The data gathering tool in this study was a self-reported questionnaire (Additional file 1), which was designed according to the existing measures in scientific literature [32–34]. The study instrument was translated from English to Persian using a standard forward-backward translation technique [35]. The original instrument was translated by a bilingual specialist. The Persian version was then retranslated into English by two independent bilingual professionals to assess retention of the original meaning in the source language. Subsequently, translators worked separately in the translation process and then prepared the final version of the Persian translation. Content validity of The Persian version of questionnaire was evaluated by a panel of experts such as 3 nutrition specialists, 3 health education specialists, and 2 instrument designers. After receiving their comments, crucial revisions were conducted in the study tool. Finally, validity of the study instrument was confirmed. The present questionnaire including four following sections:

#### General characteristics

The first part contains personal information such as age, gender, weight, height, field of study, student education, father’s education, mother’s education, father job, mother’s job, ethnicity, marital status, participating in nutrition educational classes, students’ monthly income, family’s monthly income, housing status, information resource for healthy nutrition.

#### Constructs of the TPB

The second part contains questions about the constructs of the theory of planned behavior (attitude, subjective norms, perceived behavioral control and behavioral intention). In general, attitudes, subjective norm and perceived behavioral control of students were measured using indirect items. The internal reliability of all subscales of the TPB variables was good, with a Cronbach’s alpha of 0.852.

### Attitude toward fast food use

The attitude of the people was evaluated using 28 indirect items (14 items of behavioral beliefs, 14 items of expectations evaluation) based on five-point the Likert scale (from strongly agree to strongly disagree) or (from very important to not at all important), and the score of each item varied from 1 to 5. The minimum and maximum score for the attitude subscale was 14 and 350, respectively. The internal reliability of attitude subscale was good, with a Cronbach’s alpha of 0.778.

### Subjective norm

Subjective norms of students were measured by 10 indirect items (5 items of normative beliefs, 5 items of motivation to comply) based on five-point the Likert scale (from strongly agree to strongly disagree) or (from very important to not at all important), and the score of each item varied from 1 to 5. The minimum and maximum score for the subjective norm subscale was 5 and 125, respectively. The internal reliability of subjective norm subscale was good, with a Cronbach’s alpha of 0.726.

### Perceived behavioral control

Perceived behavioral control were measured by 18 indirect items (9 items of control beliefs, 9 items of perceive power) based on five-point the Likert scale (from strongly agree to strongly disagree) or (from extremely difficult to extremely easy), and the score of each item varied from 1 to 5. The minimum and maximum score for the perceived behavioral control subscale was 9 and 225, respectively. The internal reliability of subscale of perceived behavioral control was good, with a Cronbach’s alpha of 0.815.

### Behavioral intention

Behavioral intention was evaluated by 8 items based on five-point the Likert scale (from strongly agree to strongly disagree), and the score of each item varied from 1 to 5. The minimum and maximum score for the Behavioral intention subscale was 8 and 40, respectively. The internal reliability of behavioral intention subscale was good, with a Cronbach’s alpha of 0.821.

#### Knowledge of participants

And the third and fourth parts are items related to food knowledge and fast food behavior. Students’ knowledge of fast food was evaluated by 14 items, and the score of each item varied from 0 to 2. The minimum and maximum score for the knowledge subscale was 0 and 28, respectively. The internal reliability of students’ knowledge was good, with a Cronbach’s alpha of 0.783.

#### Fast food use

Students’ fast food consumption was assessed by frequency of use in a past month. The term “Fast food” was defined as hamburgers, doughnuts, hot dog, snack, pizza, fried chicken and fried potatoes. The frequency of fast food use was analyzed for each food category.

### Statistical analyses

All statistical analyzes were performed using SPSS 16.0 software. Descriptive statistics methods such as frequencies, means and standard deviations were used along with independent t and χ2 tests. Pearson correlation test was used to investigate the relationship between TPB variables with intent to use fast food and the real use of fast food. Multiple regressions were used for further analysis.

## Results

### Descriptives

A total of 330 students were selected and recruited to the study, but some subjects (31 samples) were excluded from the study due to incomplete questionnaires (21cases), and no return of questionnaires (10 cases). Statistical analyses were performed on 229 students. Of these, 28.4% of the students were males and 71.6% were females. The results of the study showed that the average age for all the students was 22.10 ± 3.30 (the average age for male and female sexes were 22.66 ± 4.47 and 21.84 ± 2.50, respectively). The two sexes differed in terms of BMI, so that the mean of BMI was higher in boy students than in girls, and this difference was statistically significant. Almost more than 72% of the students had normal weight, and 28% of subjects were in other weights. Approximately 20.51, 54.50, 79.77% of the students reported the professional doctoral degree, Azeri ethnicity and single.

In addition, findings revealed that 64.90% of the participants lived in the dormitory, and 35.10% of them lived in personal or rental housing. The most common level of education for father (37.10%) and mother (44.10%) of students was diploma. Nearly, 46.50% of students gained food information (especially fast food) from health care providers, while 53.50% of them received their food information from other sources. Most students had zero monthly income, but 61.61% of the students reported their family’s monthly income more than 50 million Rials and 38.39% of their family had income lower than the mentioned amount. Table 1 provides detailed information on students’ characteristics.

**Table 1:** The characteristics of the study sample

Variable	The whole group	Sex		χ2 value	***p***.value
		Male(85) N (%)	Female(214) N (%)		
**Body weight status**				20.44	**< 0.001**
Underweight (less than 18.5)	23 (7.70)	2 (0.33)	21 (7.01)		
Normal (between 18.5 and 24.9)	216 (72.20)	53 (17.70)	163 (54.94)		
Overweight (Between 25 and 29.9)	52 (17.40)	25 (8.35)	27 (9.01)		
Obese (more than 29.9)	8 (2.70)	5 (1.66)	3 (1.00)		
**Student education level**				1.50	0.47
Bachelor	142 (47.50)	37 (12.37)	105 (35.13)		
Masters	4 (1.30)	2 (0.67)	2 (0.67)		
The professional doctor	153 (51.20)	46 (15.36)	107 (35.80)		
**Ethnicity**				0.65	0.72
Turkish	163 (54.50)	46 (15.36)	117 (39.22)		
Kurdish	98 (32.80)	30 (10.02)	68 (22.72)		
Other	38 (12.70)	9 (3.00)	29 (9.68)		
**Marital status**				0.01	0.91
Single	238 (79.60)	68 (22.72)	170 (56.92)		
Married	61 (20.40)	17 (5.67)	44 (14.69)		
**Housing**				0.63	0.72
Personal home	88 (29.40)	23 (7.68)	65 (21.71)		
Dorm	194 (64.90)	58 (19.37)	136 (45.42)		
Rented home	17 (5.70)	4 (1.33)	13 (4.34)		
**Mother’s education level**				9.71	**0.04**
Illiterate	46 (15.40)	20 (6.68)	26 (8.68)		
Diploma and under diploma	132 (44.10)	28 (9.35)	104 (34.73)		
Bachelor	62 (20.70)	20 (6.68)	42 (14.02)		
Masters	46 (15.40)	12 (4.00)	34 (11.35)		
Doctor	13 (4.30)	5 (1.67)	8 (2.67)		
**Father’s education level**				14.5	**0.007**
Illiterate	30 (10.00)	16 (5.34)	14 (4.67)		
Diploma and under diploma	111 (37.10)	22 (7.34)	89 (29.72)		
Bachelor	77 (25.80)	22 (7.34)	55 (18.37)		
Masters	60 (20.10)	17 (5.67)	43 (14.36)		
The doctor	21 (7.00)	8 (2.67)	13 (4.34)		
**Father’s job**				3.59	0.46
Worker	7 (2.30)	4 (1.33)	3 (1.00)		
Employee	122 (40.80)	32 (10.68)	90 (30.06)		
Unemployed	27 (9.00)	9 (3.00)	18 (6.01)		
Free job	134 (44.80)	37 12.35)	97 (32.39)		
The doctor	9 (3.00)	3 (1.00)	6 (2.00)		
**Mother’s job**				3.45	0.48
Worker	6 (2.00)	3 (100)	3 (1.00)		
Employee	67 (22.40)	19 (6.34)	48 (16.06)		
Housewife	96 (32.10)	22 (7.34)	74 (24.71)		
Free job	123 (41.10)	39 (13.02)	84 (28.05)		
The doctor	7 (2.30)	2 (0.67)	5 (1.67)		
**Participate in nutrition education class**				1.71	0.19
Yes	106 (35.50)	35 (11.69)	71 (23.71)		
No	193 (64.50)	50 (16.70)	143 (47.76)		
**The source of nutritional information**				6.61	0.16
Health care personnel	139 (46.50)	40 (13.36)	99 (33.06)		
Family and friends	47 (15.70)	13 (4.34)	34 (11.35)		
Radio and TV	48 (16.10)	13 (4.34)	35 (11.69)		
Book, magazine and newspaper	33 (11.00)	5 (1.67)	28 (9.35)		
Other	32 (10.70)	14 (4.67)	18 (6.01)		
**Student monthly income**				17.21	**0.002**
Zero	238 (79.60)	56 (18.70)	182 (60.78)		
Less than 2000,000 Rials	22 (7.40)	12 (4.00)	10 (3.34)		
Between 2000,000 and 3499,000Rials	10 (3.30)	5 (1.67)	5 (1.67)		
Between 3500,000 and 5000,000 Rials	9 (3.00)	2 (0.67)	7 (2.30)		
More than 5000,000 Rials	20 (6.70)	10 (3.34)	10 (3.34)		
**Family monthly income**				10.31	**0.015**
Less than 20,000,000 Rials	37 (12.40)	18 (6.01)	19 (6.34)		
Between 20,000,000 and 34,990,00 0Rials	50 (16.70)	9 (3.00)	41 (13.69)		
Between 35,000,000 and 50,000,000Rials	28 (9.40)	7 (2.30)	21 (7.01)		
More than 50,000,000 Rials	184 (61.50)	51 (17.03)	133 (44.42)		

### Main analysis

Table 2 presents the mean score of knowledge and variables of the study-related theoretical framework. As the mean score of subjective norm, perceived behavioral control and behavioral intention in male students compared to female students was high, but those were not significant statistically(*p* > 0.05).

**Table 2:** The mean score of knowledge and the constructs of the TPB among students in terms of sex

Variable	The whole group	Sex		t value	***p***.value
		Male	Female		
	^a^M ± SD^b^	M ± SD	M ± SD		
Knowledge	21.68 ± 5.26	21.15 ± 5.06	21.89 ± 5.33	1.09	0.27
Attitude	190.30 ± 43.54	188.42 ± 40.08	191.06 ± 44.90	0.47	0.63
Subjective norm	55.15 ± 13.10	55.69 ± 13.51	54.93 ± 12.96	−0.44	0.65
Perceived behavioral control	98.13 ± 36.52	101.61 ± 39.80	96.75 ± 35.14	−1.04	0.30
Behavioral intention	27.96 ± 6.19	28.21 ± 6.86	27.86 ± 5.92	−0.43	0.66
Fast-food consumption	2.70 ± 3.91	2.97 ± 4.58	2.57 ± 3.61	−0.79	0.43

^a^ Mean, ^b^ Standard Deviation

Some variables of the TPB were significantly correlated with each other (*P* < 0.01, Table 3). In particular, fast food consumption behavior was highly (*r* = 0.382) correlated with behavioral intention. Multiple regression analyses were conducted to determine the relative importance of the variables of the TPB to behavioral intention and fast food consumption behavior (Tables 4 and 5). In these analyzes, when the attitude toward behavior, subjective norms, and perceived control was regressed to behavioral intention, the model was very significant (*P* = 0.000) and explained 0.347 of variance of behavioral intention. While attitude and perceived behavioral control were not significant, the subjective norms and students’ knowledge were significantly related to the intention to eat fast food. It seems that subjective norms and students’ knowledge to be the most important predictors of behavioral intent. Table 4 shows more information about predictors of behavioral intention.

**Table 3:** Correlation matrix of variables of the study theory (TPB)

Variable	Behavioral intention	Fast-food consumption	Knowledge	Attitude	Subjective norm	Perceived behavioral control
Behavioral intention	1.00					
Fast-food consumption	.382**	1.00				
Knowledge	.355**	.234**	1.00			
Attitude	.068	.215**	−.111	1.00		
Subjective norm	.351**	.055	.073	.292**	1.00	
Perceived behavioral control	.037	.291**	−.276**	.539**	.325**	1.00

** *p* < 0.01

**Table 4:** Multiple linear regression analysis on students’ behavioral intention

Model 1	Unstandardized Coefficients		Standardized Coefficients	t-value	***p***-value	R^**2**^
	B	Std. Error	Beta			
Constant	10.582	2.222		4.762	.000	0.347
Knowledge	.400	.064	.340	6.292	.000	
Attitude	.000	.009	−.002	−.038	.970	
Subjective norm	.005	.011	.318	5.726	.000	
Perceived behavioral control	.150	.026	.029	.441	.659

**Table 5:** Multiple linear regression analysis on students’ fast food consumption behavior

Model2	Unstandardized Coefficients		Standardized Coefficients	t-value	***p***-value	R^**2**^
	B	Std. Error	Beta			
Constant	2.804	1.556		1.802	.073	0.231
Knowledge	.094	.046	.127	2.061	.040	
Attitude	.009	.006	.095	1.456	.146	
Subjective norm	.003	.019	.010	.161	.872	
Perceived behavioral control	.022	.007	.205	2.967	.003	
Behavioral intention	.013	.002	.215	3.155	.000	

The second model, using fast food consumption as a dependent variable, was also very significant (*P* = 0.000), and explained nearly a quarter of the variance (0.231) of fast food consumption. Both behavioral intention and perceived behavioral control were significantly associated with fast food consumption, of which behavioral intention appeared to be more important. Table 5 presents more information about predictors of fast food consumption.

## Discussion

This investigation was conducted on a sample of university students to assess the status of their fast-food consumption. It also examined the factors affecting behavioral intent and fast food consumption by applying the TPB. The results of the present study showed that students consumed fast food at an average of 2.7 times a month. Fast food in male students was often reported more than female students. A study on fast food consumption among students at Daejeon School reported monthly frequencies of fast food types: 2.7 for burgers, 2.1 for French fries, 1.8 for chicken [24]. Results of Kim study and other similar researches [31, 36] approximately were in line with findings of the present study.

Given that most men do not have the time and skill to make traditional foods, and because of a lot of work, they prefer to turn to fast-foods, and so they are more likely to use fast foods. Meanwhile, the results of some studies indicate that most women are not very happy from high weight and are more likely to reduce their weight [37]. Therefore women do not have a positive attitude toward obesogenic foods compared to men [38], which can be a reason for consuming less fast food among women. Instead, the results of a study done by Seo et al. In Korea indicated that fast food consumption among high school students was 4.05 times a month and this consumption was reported among boys more than girls [28]. The results of the Korean study were contrary to the results of the study, meaning that fast food in Korean samples was more than Iranian. The reason for this difference can be traced to factors such as sample size, cultural, social, and economic characteristics of the samples.

Performing and not performing the behavior by a person is a function of several factors based on the theory of planned behavior. One of these factors is the person’s intention and desire to do the behavior. Behavioral intention itself is also affected by factors such as attitude, students’ knowledge, social pressure, and perceived behavioral control. In the present study, based on linear regression analysis, students’ knowledge and social pressure were both related to their intention and consume fast foods. That is, students who had the necessary information about nutrition, especially fast foods, had a high intent to choose and consume foods.

Several studies have examined the relationship between knowledge of foods and their contents and attitudes toward fast foods and processed foods or relationship between attitudes toward food additives and food choice behavior [39–42]. Aoki et al. [39] found that information about food and its contents positively or negatively affects attitudes and intentions towards food. They pointed out that food information was important for consumers in choosing food. Back and Lee [43] found that consumers had inadequate and incorrect information about foods, which could affect their attitudes or intent. These studies suggest that providing more information about foods and their compounds can help them to improve their attitude towards foods. Therefore, training on the performance, benefits and safety of foods, including positive and negative sides, should prevent misunderstandings about food supplements and reduce food safety concerns.

The findings of the present investigation showed that subjective norms of students were effective on intent to use fast foods. Friends had the most impact on the plan to eat fast foods, as expected. In addition, the normative beliefs of students were also more positive for friends than family and teachers. This conclusion suggests that most training programs should focus on their friends as a critical group that may affect intent to use fast foods.

Results of some previous studies were similar to findings of the current study. One study conducted by Mirkarimi et al. highlighted that subjective norms had the main role on students’ intent to use fast foods [44]. In the other words, they found that behavioral intention was affected by subjective norms. In addition, the study of Yarmohammadi and et al. showed that subjective norms predict intention and behavior [31].

In this study, TPB demonstrated to be a sound conceptual framework for explaining closely35% of the variance in students’ behavioral intention to consume fast-food. Among the TPB variables, subjective norm and knowledge of students were the most important predictors of intention to use fast foods. These findings are consistent with other results that identify that subjective norms have a significant effect on consuming fruits and vegetables [45]. In study of Lynn Fudge, Path analysis highlighted that TPB explained adolescent fast-food behavioral intention to consume fast food. The model identified subjective norms had the strongest relationship with adolescent behavioral intention to consume fast food [46].

The results of this study showed that the attitude toward fast food behavior did not predict intent and the behavior. However, some studies have reported contradictory findings with the study. For example, the findings of Stefanie and Chery’s study showed that attitude was a predictor for intent to use healthy nutrition [47]. Yarmohammadi and colleagues stated in their study that attitude was the most important predictor of behavioral intent [31]. In the study of determinants of fast food intake, Dunn et al. has identified attitude as a predictor of the intent of fast food consumption [32]. The results of studies by Seo et al., Ebadi et al., along with the findings of this study, showed that attitude toward fast food consumption is not significantly related to behavioral intention [28, 48]. Based on the findings of the current study, fast-food consumption of students was also influenced by some the TPB variables. Multiple linear regression analyses revealed that the constructs of the TPB explained fast food use behaviors with R-squared (R^2^) of 0.23. In these analyses, intention, perceived behavioral control, and knowledge were known as effective factors on fast-food consumption. Among the TPB constructs, behavioral intention was the most important predictor of fast-food consumption. The intention plays a fundamental role in the theory of planned behavior. The intentions include motivational factors that influence behavior and show how much people want to behave and how hard they try to do the behavior [49]. In study Ebadi et al., regression analysis showed the intention as a predictor of fast food consumption behavior [48]. In studies of Stefanie et al. and Seo et al., has reported intention as correlate of the behavior [28, 47]. All these studies confirmed and supported this part of our study findings. In addition, the results indicated that perceived behavioral control directly influenced the behavior of fast-food consumption. Some investigations confirmed this portion of our results. For instance, the results of Dunn et al. showed that perceived behavioral control (PBC) and intent predicted the behavior of fast food consumption [32]. Also, in the study of Seo et al., regression analysis showed that fast food consumption behavior was correlated with perceived behavioral control [28]. Yarmohammadi et al. found that in predicting behavior, perceived behavioral control along with intention could predict 6% of behavior [31]. Although this study provides valuable knowledge regarding the relationships between behavioral intent and TPB variables, this study, like other studies, has a number of limitations. First, a cross-sectional study was used to examine the relationship between the variables. Due to the fact that in cross-sectional studies, all data are collected in a period of time, as a result, these studies do not have the necessary ability to examine the cause-and-effect relationships between variables. Second, the results of this type of study can only be generalized to populations with similar characteristics and have no generalizability beyond that. Third, since the data of this study were collected using the self-report questionnaire, the respondents may have errors and bias in completing the questionnaire and this can affect the results of the study.

## Conclusions

In sum, this study was conducted to identify factors influencing intention and behavior of fast-food consumption among students by using the theory of planned behavior. The findings revealed that changeability of students’ intention to use fast food and their real behavior is dependent on the TPB variables. As this theoretical framework explained 35, 23% of intent to consume fast-foods and fast-food consumption, respectively. Among the TPB constructs, knowledge and subjective norm were known as the most important predictors of intention to use fast foods. In addition, the results indicated that intention and perceived behavioral control were the most important factors influencing consumption of fast foods among participants. It is imperative that health educators and promoters use these results in designing suitable educational interventions to improve people’s nutritional behavior.

## Supplementary Information

**Additional file 1.** The questionnire used in the study to collect the data. The first part of the questionnaire included General characteristics. The second part of the questionnaire consisted of the Constructs of TPB. The third part consisted of knowledge of participants. The fourth part consisted of Fast food use.

## Data Availability

The datasets generated during and/or analyzed during the current study are not publicly available due to confidentiality of data and subsequent research, but are available from the corresponding author on reasonable request.

## Abbreviations

TPB: Theory of Planned Behavior
TRA: Theory of Reasoned Action
SPSS: Statistical Package for Social Sciences
BMI: Body Mass Index
