# Supplementary material for: Determining intention, fast food consumption and their related factors among university students by using a behavior change theory
Source: BMC Public Health. 2022 Feb 15;22:314. doi: 10.1186/s12889-022-12696-x (PMC8845370; doi:10.1186/s12889-022-12696-x)
Supplement: Supplementary file 1 — Additional file 1. The questionnire used in the study to collect the data. The first part of the questionnaire included General characteristics. The second part of the questionnaire consisted of the Constructs of TPB. The third part consisted of knowledge of participants. The fourth part consisted of Fast food use. [file 12889_2022_12696_MOESM1_ESM.pdf]

Questionnaire number ....

Dear student

This questionnaire has been designed to evaluate the attitude and factors affecting the consumption of fast foods, including a variety of sandwiches (sausages, sausages), a variety of pizzas, hot dogs, fried chicken with fries, snacks (salty, sweet and fatty) Burgers, a food donut that is a favorite of many young people. Please do not write your first and last name on the questionnaire, this will help you answer the questions with complete freedom and honesty. All the information collected in this study will be analyzed in general and its results will be used to promote the health of your loved ones and other people in the community. We hope to be able to take steps to solve some of the country's nutritional problems and improve the health of people in our community.

Many thanks for your cooperation

"Please answer all questions even if you are not sure about the answer"

Section A. Demographic characteristics

1- Age (by year) ………
   Height (in centimeters): .... Weight (in kilograms): ....

Gender: a) Female b) Male

1- Level of education: a) Bachelor's degree b) Master's degree c) Professional doctorate (medicine, dentistry, pharmacy)
2- Mother's education level: a) illiterate b) diploma c) bachelor's degree c) master c) doctorate
3- Father's education level: a) illiterate b) diploma c) bachelor's degree c) master c) doctorate
4- Father's job: a) Worker b) Employee c) Unemployed c) Freelance h) Doctor x) Other cases
5- Mother's job: a) Worker b) Employee c) Unemployed c) Freelance h) Doctor x) Other cases

6- Ethnicity: a) Azeri b) Kurd c) Other cases
7- Marital status: a) single b) married
8- Occupation: a) Employed b) Unemployed
9- Your monthly income:
A) Under 2000,000 Rials b) 200,0000-350,0000 Rials C) 3500,000- 5000,000 Rials d) Above 5000,000 Rials e) I have no income
10- Monthly family income:
A) Under 20000,000 Rials b) 20000,000-35000,000 Rials C) 35000,000- 50000,000 Rials d) Above 50000,000 Rials
11- Did you have a formal training class on proper nutrition? 1) Yes 2) No
12- Which of the following sources do you prefer to learn more about proper nutrition?
A) Physician and nutritionist b) Family members, friends and acquaintances c) Radio and television d) Books, magazines and newspapers e) Other cases

15-Specify your residence status.

A) Private house b) Dormitory c) Rented house with friends

Section B

Dear student, please read the following questions and mark (✵) in front of the correct answer.

1) Do fast foods have any side effects for the body? A) Yes b) No c) I do not know

2) Is careful control of eating and eating healthy foods important? A) Yes b) No c) I do not know 3) If we consume a lot of fast food foods, the diseases caused by consuming them usually appear on the same day? A) Yes b) No c) I do not know

4) Does proper and healthy nutrition play a role in the incidence of chronic diseases?

A) Yes b) No c) I do not know

5) Do we have to limit the consumption of fast foods to have a healthy life?

A) Yes b) No c) I do not know

6) Does the constant use of fast food in the restaurant environment cause you to stay away from the family environment? A) Yes b) No c) I do not know.

7) Does the packaging of fast food (use of plastic materials) have an impact on the environment? A) Yes b) No c) I do not know

8) Is the cause of obesity in most children, adolescents and young people today, especially in developing countries, eating unhealthy foods such as fast food?

A) Yes b) No c) I do not know

9) Is salt, oil and sugar used properly in the preparation of fast foods?

A) Yes b) No c) I do not know

10) By consuming fast food in every meal, is the body's need for the food groups needed by the body fully met? A) Yes b) No c) I do not know

11) Do fast food restaurants change the oil used for each fast food preparation?

A) Yes b) No c) I do not know

12) Are the ingredients used in the preparation of fast foods instead of meat, such as sausages, useful for the body? A) Yes b) No c) I do not know

13) Does regular consumption of fast food, especially in children, increase the risk of diseases other than obesity and nutritional problems such as allergies and asthma?

A) Yes b) No c) I do not know

14) Are preservatives used in raw materials for fast food (including sausages)?

A) Yes b) No c) I do not know

Section C

The questions in this section of the questionnaire assess the "attitude" towards fast food consumption.

1) Fast food is delicious.

A) strongly agree b) agree c) Do not know d) disagree e) strongly disagree

2) Eat delicious food for me ....

A) extremely important b) important c) I have no idea d) unimportant e) extremely unimportant

3) I feel satisfied after eating fast food.

A) strongly agree b) agree c) Do not know d) disagree e) strongly disagree

4) Feeling satisfied after eating for me ....

A) extremely important b) important c) I have no idea d) unimportant e) extremely unimportant.

5) Eating fast food (3 or more times a week) is good for health.

A) strongly agree b) agree c) Do not know d) disagree e) strongly disagree

6) Maintaining the health of the body for me ....

A) extremely important b) important c) I have no idea d) unimportant e) extremely unimportant.

7) I think eating fast food (3 or more times a week) probably causes weight gain.

A) strongly agree b) agree c) Do not know d) disagree e) strongly disagree

8) Keeping the weight in a normal size for me ....

A) extremely important b) important c) I have no idea d) unimportant e) extremely unimportant

9) Eating fast food (3 times or more a week) is very convenient for me.

A) strongly agree b) agree c) Do not know d) disagree e) strongly disagree

10) Having a comfortable food source for me ....

A) extremely important b) important c) I have no idea d) unimportant e) extremely unimportant

11) Eating fast food (3 or more times a week) reduces the amount of work I have to do to prepare food (for example, planning, preparing and cleaning).

A) strongly agree b) agree c) Do not know d) disagree e) strongly disagree

12) Reducing the amount of work I have to do to prepare food (for example, planning, preparing and cleaning) for me ....

A) extremely important b) important c) I have no idea d) unimportant e) extremely unimportant

13) Eating fast food (3 or more times a week) saves me time.

A) strongly agree b) agree c) Do not know d) disagree e) strongly disagree

14) Saving time spent preparing food for me ....

A) extremely important b) important c) I have no idea d) unimportant e) extremely unimportant

15) Eating fast food gives me the opportunity to eat my food wherever I want (for example in a restaurant, car and home).

A) strongly agree b) agree c) Do not know d) disagree e) strongly disagree

16) To be able to eat wherever I want ....

A) extremely important b) important c) I have no idea d) unimportant e) extremely unimportant

17) Eating fast food (3 or more times a week) is relatively cheap compared to restaurant food.

A) strongly agree b) agree c) Do not know d) disagree e) strongly disagree

18) Cheap food for me ....

A) extremely important b) important c) I have no idea d) unimportant e) extremely unimportant

19) Eating (3 or more times a week) in fast food restaurants allows me to get out of the house.

A) strongly agree b) agree c) Do not know d) disagree e) strongly disagree

20) Leaving home for any reason for me ....

A) extremely important b) important c) I have no idea d) unimportant e) extremely unimportant

21) Eating fast food (3 or more times a week) reduces the possibility of eating traditional foods with the family

A) strongly agree b) agree c) Do not know d) disagree e) strongly disagree

22) Maintain opportunities to eat traditional food with my family ....

A) extremely important b) important c) I have no idea d) unimportant e) extremely unimportant

23) Eating fast food (3 or more times a week) causes habituation.

A) A) strongly agree b) agree c) Do not know d) disagree e) strongly disagree

24) Constant consumption of fast food for me ....

A) extremely important b) important c) I have no idea d) unimportant e) extremely unimportant

25) Eating fast food (3 times or more per week) causes inactivity.

A) strongly agree b) agree c) Do not know d) disagree e) strongly disagree

26) A sedentary and sedentary life for my health ....

A) extremely important b) important c) I have no idea d) unimportant e) extremely unimportant

27) Eating fast food (3 or more times a week) is harmful to the environment.

A) strongly agree b) agree c) Do not know d) disagree e) strongly disagree

28) Environmental protection for me ....

A) extremely important b) important c) I have no idea d) unimportant e) extremely unimportant

Section D

The questions in this section of the "Perceived Behavioral Control" questionnaire are assessed.

1) My lifestyle is such that I will have little free time next month.

A) strongly agree b) agree c) Do not know d) disagree e) strongly disagree

2) Having less free time causes me to eat fast food 3 or more times a week.

A) strongly agree b) agree c) Do not know d) disagree e) strongly disagree

3) I feel guilty about eating certain foods.

A) strongly agree b) agree c) Do not know d) disagree e) strongly disagree

4) Feeling guilty after eating fast food prevents me from eating fast food (3 times or more a week).

A) strongly agree b) agree c) Do not know d) disagree e) strongly disagree

5) Sometimes I want to eat some food.

A) strongly agree b) agree c) Do not know d) disagree e) strongly disagree

6) My desire to eat fast food makes me eat fast food often (3 times or more a week).

A) strongly agree b) agree c) Do not know d) disagree e) strongly disagree

7) Most of the time I only eat.

A) strongly agree b) agree c) Do not know d) disagree e) strongly disagree

8) Eating alone allows me to eat fast food frequently (3 times or more a week).

A) strongly agree b) agree c) Do not know d) disagree e) strongly disagree

9) I can not prepare food for myself, because I do not know how to cook.

A) strongly agree b) agree c) Do not know d) disagree e) strongly disagree

10) Because I do not know how to cook, I prefer to eat fast food frequently (3 times or more a week).

A) strongly agree b) agree c) Do not know d) disagree e) strongly disagree

11) I have to be careful about the foods I eat because of my weight.

A) strongly agree b) agree c) Do not know d) disagree e) strongly disagree

12) Worrying about my weight prevents me from eating fast food (3 times or more a week).

A) strongly agree b) agree c) Do not know d) disagree e) strongly disagree

13) I have to take care of what I eat for the sake of my health.

A) strongly agree b) agree c) Do not know d) disagree e) strongly disagree

14) Concerns about my health keep me from eating fast food (often or more than once a week).

A) strongly agree b) agree c) Do not know d) disagree e) strongly disagree

15) I have no access to fast food where I live.

A) strongly agree b) agree c) Do not know d) disagree e) strongly disagree

16) Lack of access to fast food, causes me not to eat fast food frequently (3 times or more per week).

A) strongly agree b) agree c) Do not know d) disagree e) strongly disagree

17) How much I eat fast food next month is more up to me.

A) strongly agree b) agree c) Do not know d) disagree e) strongly disagree

18) From next month, I can reduce the number of times I eat my fast food.

A) strongly agree b) agree c) Do not know d) disagree e) strongly disagree

Section E

The questions in this section of the questionnaire assess " subjective norms" regarding fast food consumption.

1)The people I respect by eating my fast food are ....

A) strongly agree b) agree c) Do not know d) disagree e) strongly disagree

2) People's opinion about eating fast food, for me ....

A) extremely important b) important c) I have no idea d) unimportant e) extremely unimportant

3) In general, my family members think that I should eat fast food 3 or more times a week.

A) strongly agree b) agree c) Do not know d) disagree e) strongly disagree

4) My family members' opinion about my nutrition, for me ....

A) extremely important b) important c) I have no idea d) unimportant e) extremely unimportant

5) In general, my friends think I should eat fast food 3 or more times a week.

A) strongly agree b) agree c) Do not know d) disagree e) strongly disagree

6) My friends' opinion about my nutrition, for me ....

A) extremely important b) important c) I have no idea d) unimportant e) extremely unimportant

7) Health experts think I should eat fast food 3 or more times a week.

A) strongly agree b) agree c) Do not know d) disagree e) strongly disagree

8) The opinion of health professionals about my nutrition, for me ....

A) extremely important b) important c) I have no idea d) unimportant e) extremely unimportant

9) My wife expects me to eat fast food 3 or more times a week.

A) strongly agree b) agree c) Do not know d) disagree e) strongly disagree

10) My wife's opinion about my nutrition, for me ....

A) extremely important b) important c) I have no idea d) unimportant e) extremely unimportant

Section F. ***Behavioral Intention Assessment Questionnaire***

1) I plan to eat less fast food next month.
   A) strongly agree b) agree c) Do not know d) disagree e) strongly disagree
2) I plan to use the right foods for my health in my diet in the coming weeks.
   A) strongly agree b) agree c) Do not know d) disagree e) strongly disagree
3) I will try to pay attention to the type of food I consume next month.
   A) strongly agree b) agree c) Do not know d) disagree e) strongly disagree

4) Next month, I plan to pay attention to the type of food that I pay for each meal and how it is prepared, that is, healthy foods.

A) strongly agree b) agree c) Do not know d) disagree e) strongly disagree

5) I will try to pay attention to my diet in future trips and parties.

A) strongly agree b) agree c) Do not know d) disagree e) strongly disagree

6) I plan to see a nutritionist at least once every 6 months for my diet in the coming months.

A) strongly agree b) agree c) Do not know d) disagree e) strongly disagree

7) I plan to stop eating fast food next month.

A) strongly agree b) agree c) Do not know d) disagree e) strongly disagree

8) I will try to eat more home diet next month.

A) strongly agree b) agree c) Do not know d) disagree e) strongly disagree

Section G

❋Please specify what kind of fast food you ate during the last week and when? Fast food dishes include: various types of sandwiches (sausages, sausages, burgers, dumplings, hot dogs, snacks (salty or sweet and fatty), types of pizza, fried chicken nuggets and chicken with fried potatoes, which These dishes may be served outdoors (on the way) or in a restaurant.

Type of fast food	Number of servings per day	Number of servings per week	Number of servings per month	Number of servings per year
Types of sandwiches (sausages, etc.)				
Types of burgers				
Dumplings				
Hot dog				
snack				
Types of pizzas				
Chicken nuggets (minced chicken breast is rolled in breadcrumbs and fried in oil)				
Fried chicken with fried potatoes				
